# Unfulfilled and method‐specific contraceptive preferences among reproductive‐aged contraceptive users in Arizona, Iowa, New Jersey, and Wisconsin

**DOI:** 10.1111/1475-6773.14297

**Published:** 2024-03-08

**Authors:** Megan L. Kavanaugh, Rubina Hussain, Ashley C. Little

**Affiliations:** ^1^ Research Division Guttmacher Institute New York New York USA

**Keywords:** access/demand/utilization of services, health policy/politics/law/regulation, observational data/quasi‐experiments, patient assessment/satisfaction, quality of care/patient safety (measurement), survey research and questionnaire design

## Abstract

**Objective:**

To identify characteristics associated with unfulfilled contraceptive preferences, document reasons for these unfulfilled preferences, and examine how these unfulfilled preferences vary across specific method users.

**Data Sources and Study Setting:**

We draw on secondary baseline data from 4660 reproductive‐aged contraceptive users in the Arizona, Iowa, New Jersey, and Wisconsin Surveys of Women (SoWs), state‐representative surveys fielded between October 2018 and August 2020 across the four states.

**Study Design:**

This is an observational cross‐sectional study, which examined associations between individuals' reproductive health‐related experiences and contraceptive preferences, adjusting for sociodemographic characteristics. Our primary outcome of interest is having an unfulfilled contraceptive preference, and a key independent variable is experience of high‐quality contraceptive care. We also examine specific contraceptive method preferences according to current method used, as well as reasons for not using a preferred method.

**Data Collection/Extraction Methods:**

Survey respondents who indicated use of any contraceptive method within the last 3 months prior to the survey were eligible for inclusion in this analysis.

**Principal Findings:**

Overall, 23% reported preferring to use a method other than their current method, ranging from 17% in Iowa to 26% in New Jersey. Young age (18–24), using methods not requiring provider involvement, and not receiving quality contraceptive care were key attributes associated with unfulfilled contraceptive preferences. Those using emergency contraception and fertility awareness‐based methods had some of the highest levels of unfulfilled contraceptive preferences, while pills, condoms, partner vasectomy, and IUDs were identified as the most preferred methods. Reasons for not using preferred contraceptive methods fell largely into one of two buckets: system‐level or interpersonal/individual reasons.

**Conclusions:**

Our findings highlight that avenues for decreasing the gap between contraceptive methods used and those preferred to be used may lie with healthcare providers and funding streams that support the delivery of contraceptive care.

**What is known on this topic**

- United States public health goals related to contraception have historically focused on contraceptive method use, but “contraceptive preference” as a key indicator may be more aligned with tenets of person‐centeredness.
- A growing body of work has begun to document people's contraceptive preferences, with national‐ and state‐level representative surveys identifying about ¼‐1/3 of contraceptive users indicating preferences for using different methods.
- Research has also documented that interactions with healthcare providers are key to whether individuals experience their contraceptive care to be high‐quality and person‐centered.

**What this study adds**

- Our state‐specific findings highlight that a quarter of contraceptive users have unfulfilled contraceptive preferences; healthcare providers should employ patient‐centered best practices to help people, especially young people, realize their preferences.
- Users of emergency contraception have the highest levels of unfulfilled contraceptive preferences; among those with unfulfilled preferences, pills, condoms, partner vasectomy, and IUDs are the most popular preferred methods.
- Avenues for decreasing gaps between contraceptive methods used and those preferred lie with healthcare providers prioritizing patient‐centered care and public and private funding streams supporting the delivery of contraceptive care.

## 1 INTRODUCTION

Contraception can be an important tool used by individuals to exercise reproductive autonomy, including helping them to determine if, when, and how to become pregnant. Broad evidence demonstrates the myriad benefits that contraception has brought to people, ranging across health, social, and economic outcomes.^1^, ^2^, ^3^ At the same time, contraception—specifically programs focused on compelling people to use any, or specific methods of, contraception—has also been used as a tool of reproductive oppression, with the most egregious and coercive examples of this occurring among people from historically oppressed communities.^4^, ^5^, ^6^, ^7^

Public health goals related to contraception in the United States, such as those enumerated in Healthy People 2030,^8^ have focused primarily on metrics of contraceptive use versus nonuse and identifying which methods are being used. However, recent scholarship has highlighted that focusing exclusively on whether and what contraception is being used obfuscates whether the methods being used represent those preferred by the user.^9^ Incorporating a person‐centered lens into the setting of, and tracking progress toward, public health goals related to contraception is an important step forward in aligning our program and policy efforts with helping people to achieve reproductive autonomy and guarding against supporting programs that perpetuate or replicate reproductive injustices of the past. In addition, documenting and quantifying the gap between methods used and those preferred to be used helps to clearly identify where programmatic and policy efforts should focus going forward.

To that end, a growing body of research has begun to document people's contraceptive preferences in a variety of settings, as well as factors associated with these preferences. Burke and Potter (2023) recently provided an overview of key findings from 11 studies that examined contraceptive preferences within the United States since 2000.^9^ From this commentary and subsequent research since it was published, about ¼ of reproductive‐aged contraceptive users at the national level^10^, ^11^ and about 25%–33% at the state‐level in Ohio^12^ and Wisconsin^13^ have unfulfilled contraceptive preferences. Among more specific populations rates are higher, ranging from 42% among Texas postpartum women^14^ and women veterans^15^ to 54% among a sample of community college students in Texas^16^ to 59% among individuals reporting housing insecurity in a small sample in Utah.^17^ Importantly, with the exception of the population‐level Ohio study,^12^ these studies have either focused on specific groups, narrowed the assessment of preferences to be only in the context of cost barriers, or did not examine method‐specific preferences.

Across these studies, factors associated with higher levels of alignment between methods used and those preferred include the use of longer‐acting and hormonal methods, having insurance coverage and a usual source of reproductive health care, and having high‐quality person‐centered contraceptive counseling.^11^, ^12^, ^13^, ^14^, ^15^ Sociodemographic characteristics, including income, race, age, parity, and relationship status, are all also associated with contraceptive preferences, with differences across the various study settings and populations. Broadly, socioeconomic characteristics associated with higher levels of social privilege in the United States—such as identifying as white, having been born in the United States, or being in higher income brackets—align with higher levels of having fulfilled contraceptive preferences.^11^, ^12^, ^13^, ^14^, ^16^, ^18^, ^19^ Among those not using their preferred contraception, common reasons for this include cost and affordability, provider‐level barriers, partner‐related reasons, or more individual‐level reasons around fertility desires and side effects.^12^, ^15^, ^16^, ^19^

This study aims to contribute to the growing body of work around contraceptive preferences, with specific attention on population‐representative samples of reproductive‐aged women in four select states. Among reproductive‐aged contraceptive users in these states, our objectives are to identify characteristics associated with unfulfilled contraceptive preferences, document reasons for these unfulfilled preferences, and examine how these unfulfilled preferences vary across specific method users.

## 2 METHODS

### 2.1 Data

For this analysis, we draw on baseline data from the Arizona, Iowa, New Jersey, and Wisconsin Surveys of Women (SoWs), longitudinal population‐based surveys conducted by the nonpartisan and objective research organization NORC at the University of Chicago. Baseline data were collected in Iowa between October 2018 and June 2019, while baseline data from Arizona, New Jersey, and Wisconsin were collected simultaneously between November 2019 and August 2020.

The survey design, sampling strategy, and data collection procedures for the SoWs are described in greater detail elsewhere.^20^ In summary, the SoWs are self‐administered surveys focused on sexual and reproductive health experiences and attitudes and are representative of the population of reproductive‐aged women (18–44 years) in each state. We note the use of “women” in this analysis and in the survey name; the screener item in the questionnaire asked females ages 18–44 to complete the survey and individuals self‐determined their own eligibility based on the screener wording. NORC randomly sampled households in each of these states using address‐based sampling methods enhanced with an age‐targeted list and demographic information from the American Community Survey; census tracts with a higher proportion of low‐income and non‐White populations were oversampled in each state.^20^ The response rate was 29% overall across the four states examined in this analysis, specifically 38% in Iowa, 32% in Arizona, 24% in New Jersey, and 38% in Wisconsin. To account for nonresponse, base sampling, adjustment for unknown eligibility, and household size, NORC provided sample weights using poststratification methods to represent the demographics of women aged 18 to 44 in each of the states. NORC's Institutional Review Board approved the data collection protocols. Given the de‐identified nature of the survey data shared with the research team, this secondary data analysis was exempt from further review.

### 2.2 Measures

Although identifying contraceptive preferences among both contraceptive users and nonusers of contraception is ideal, data on contraceptive preferences in this study are only available among contraceptive users, as only this group of respondents was asked about them. Respondents were eligible for this analysis if they indicated using any contraceptive method (withdrawal, birth control pills, birth control patch, vaginal ring, Depo‐Provera®, IUD, implant, male condoms, other barrier methods, fertility awareness‐based methods (FABMs), emergency contraception (EC), partner's vasectomy, or other) in the past 3 months, even if for reasons other than to prevent pregnancy. Importantly, any respondents who indicated “no” to any other methods but “yes” to a separate survey item: *Have you had a tubal ligation (“tubes tied” or “Essure”) or another operation (such as a hysterectomy) that makes you unable to get pregnant?*, were not considered to be current contraceptive users for this analysis, as those who reported using any of these permanent methods of contraception were skipped out of answering questions regarding contraceptive preferences.

Our primary outcome of interest in this analysis is having an unfulfilled contraceptive preference. Current contraceptive users were asked: *If you could use any birth control method you wanted, what method(s) would you use? Please check all that apply*. Respondents who answered either “I am using the method that I want to use” or who selected a method that they reported currently using were considered to be using their preferred contraceptive method. All other respondents were considered to have an unfulfilled contraceptive preference, with method‐specific contraceptive preferences assigned according to which method respondents indicated that they would want to use. Respondents who selected more than one preferred contraceptive method were coded to each method‐specific preference they indicated. Our full analytical sample included 4660 respondents across the four states who were currently using a contraceptive method and answered the contraceptive preference item.

We investigate associations between several independent variables and our outcome. A key independent variable of interest is respondents' experiences of high‐quality contraceptive care, determined by their responses to five questions, including whether they had received contraceptive care in the past 12 months, and four questions that make up the person‐centered contraceptive counseling (PCCC) metric. This metric draws on likert‐scale responses to four items related to respondents' most recent contraceptive provider experience (respecting the respondent as a person, letting the respondent say what mattered to them about birth control, taking the respondent's preferences about their birth control seriously, and giving the respondent enough information to make the best decision about their birth control). Following published guidance,^21^ among those who reported having received contraceptive care in the past 12 months, we created a two‐category variable assigning those who indicated “excellent” on all four items of the PCCC metric to have received “excellent care” and those who did not indicate “excellent” on all four items to have received “less than excellent care”. Respondents who reported “prefer not to answer” to all four PCCC items were excluded from the denominator.

We also examined associations between several respondent sociodemographic and reproductive health‐related characteristics and our outcome of interest. These characteristics include age, race, ethnicity, nativity, sexual orientation, educational attainment, employment status, income as a percentage of the federal poverty level, relationship status, health insurance coverage, sexual activity, and use of a provider‐involved method. This last variable is a binary categorization of those methods that typically require a healthcare provider interaction for either initiation (i.e., implant or IUD) or ongoing use (i.e., oral‐contraceptive pill, patch, vaginal ring, and shot) versus those that typically do not require provider interaction (i.e., condom, withdrawal, partner vasectomy, barrier methods, FABMs, EC, or another method reported via write‐in). The survey item did not explicitly distinguish between the types of EC, so we made an analytic decision to broadly categorize EC in this analysis as not requiring provider interaction, although some forms of EC require provider interaction via prescription or insertion. NORC performed hot deck imputation on missing information for measures of age, race/ethnicity, nativity, educational attainment, employment status, relationship status, and income, and we used these imputed versions of these measures for this analysis.

Among respondents who reported having unfulfilled contraceptive preferences, we examined responses to the question: *What is the main reason you are not currently using the birth control method you want to use*? This survey item provided a list of 11 reason response options in Iowa and 19 in Arizona, New Jersey, and Wisconsin, with the additional eight reasons in the three latter states being expansions of individual options in Iowa. All surveys included an open‐ended response option; write‐in responses were recoded to an existing option or grouped into a newly created reason category. As a team, we decided to present reasons for not using one's preferred method in four broad categories to capture the diversity of response options available across the questionnaires: system level reasons, interpersonal/individual level reasons, other reasons, and not sure. Each of these categories included reasons specifically offered as options in each version of the questionnaire. “System level reasons” referred to policy, insurance, or clinic reasons that could have prevented the respondent from receiving their preferred method, often out of the respondent's own control. “Interpersonal/individual level reasons” included reasons that stem from respondents' own individual behaviors, experiences, or plans for their reproductive health.

### 2.3 Statistical analyses

We first conducted descriptive analyses of the analytic sample of current contraceptive users by selected sociodemographic characteristics, separately by state and also pooled across states. Again separately by state and pooled across the four study states, we examined percentages who reported unfulfilled contraceptive preferences by respondent characteristics and, for the pooled sample, we conducted Pearson χ^2^ tests to determine significant differences in this outcome by each characteristic. Next, pooling across the four states, we ran a series of two multivariable logistic regression models to estimate adjusted marginal effects, or the average effect of respondent characteristics on the probability of the outcome of unfulfilled contraceptive preferences, with specific attention to the role played by respondents' experiences of high‐quality contraceptive care. The first model included all sociodemographic characteristics examined in this study as well as a variable to account for variation across the four state settings, while the second set of models was narrowed to respondents who had received contraceptive care in the past 12 months (*N* = 2302) and included all of the same variables as the first model with the addition of the PCCC variable. We highlight adjusted marginal effects (aMEs) of associations and their 95% confidence intervals (CIs) in models significant at, or close to, the *p* < 0.05 level.

For all remaining analyses, we drew on data pooled across the four study states due to the smaller subsample of those reporting unfulfilled contraceptive preferences and of those using specific methods in each state. Among those respondents with unfulfilled contraceptive preferences, we calculated the prevalence of reporting each reason group for not using one's preferred contraceptive method and the prevalence of reporting each specific method preferred. Finally, we calculated the prevalence of unfulfilled contraceptive preferences according to each method currently used. All analyses were based on weighted data and completed using the svy command in Stata 18.0 to account for the complex sampling designs.

## 3 RESULTS

### 3.1 Respondent characteristics, overall and among those with an unfulfilled contraceptive preference

Our analytical sample is comprised of 4660 respondents across Arizona, Iowa, New Jersey, and Wisconsin (Table 1). We include state‐level demographics in Table 1, but we report here in the text primarily on the sample pooled across states. About half of the pooled sample (48%) was aged 18–29, 19% identified as non‐white or multiracial/other and 19% identified as Hispanic. The majority of respondents in each state and across states reported being straight, having at least some education beyond high school, being employed, living at 200% or above the federal poverty level, being married or cohabiting, being sexually active, and having private health insurance. Most respondents reported current use of a provider‐involved contraceptive method, having received contraceptive care in the past year and, among those who got this care, that it had been excellent person‐centered contraceptive care.

**TABLE 1:** Percent distribution of current contraceptive users in Arizona, Iowa, New Jersey, and Wisconsin, overall and among those with an unfulfilled contraceptive preference, by selected sociodemographic and contraceptive use characteristics, 2019–2020.

	Pooled across states			Arizona		Iowa		New Jersey		Wisconsin	
	Contraceptive users (*N* = 4660)	Contraceptive users with an unfulfilled contraceptive preference (*N* = 917)		Contraceptive users (*N* = 1045)	Contraceptive users with an unfulfilled contraceptive preference (N = 228)	Contraceptive users (*N* = 1406)	Contraceptive users with an unfulfilled contraceptive preference (*N* = 236)	Contraceptive users (*N* = 1102)	Contraceptive users with an unfulfilled contraceptive preference (*N* = 246)	Contraceptive users (*N* = 1107)	Contraceptive users with an unfulfilled contraceptive preference (*N* = 207)
	Weighted %	Weighted %	*p* value^a^	Weighted %	Weighted %	Weighted %	Weighted %	Weighted %	Weighted %	Weighted %	Weighted %
Total	100	23		100	25	100	17	100	26	100	20
Age											
18–24	27	**25**	**0.020**	30	26	31	19	24	27	26	27
25–29	21	**24**		21	24	19	18	21	35	22	15
30–34	18	**22**		18	23	18	15	19	23	17	24
35–39	18	**25**		16	31	17	19	20	29	19	17
40–44	15	**16**		14	21	15	15	15	13	15	15
Race											
White	81	**21**	**0.010**	80	23	93	17	71	23	90	20
Black	6	**36**		3	30	2	9	10	44	6	26
Asian/Pacific Islander	5	**24**		3	40	2	19	10	19	2	34
American Indian or Alaska Native	1	**32**		3	30	0	0	1	56	0	0
Multiracial/other	6	**27**		10	28	2	26	8	27	2	22
Ethnicity											
Not Hispanic	81	**22**	**<0.01**	62	23	97	17	81	24	95	20
Hispanic	19	**30**		38	29	3	29	19	31	5	27
Nativity											
Born in the United States	85	**22**	**<0.01**	84	23	94	17	76	25	94	20
Born outside of the United States	15	**31**		16	38	6	22	24	30	6	23
Sexual orientation											
Straight	90	**22**	**<0.01**	88	25	89	16	92	25	89	19
Not straight^b^	10	**31**		12	26	11	26	8	41	11	32
Lesbian or Gay	0	0		0	0	1	0	0	0	0	0
Bisexual	7	31		8	28	6	29	5	39	7	30
Pansexual	2	35		2	15	2	23	1	64	1	45
Queer/Other	2	33		1	29	1	37	2	35	2	32
Educational attainment											
High school graduate, GED, or less	15	**25**	**0.040**	19	25	12	18	13	24	14	31
Some college or associate degree	44	**25**		50	27	49	19	34	33	45	19
College graduate or more	42	**20**		31	22	39	16	53	22	40	17
Employment^c^											
Employed	76	**21**	**<0.01**	70	22	80	17	76	23	81	19
Unemployed	5	**28**		3	39	13	20	4	40	3	8
Out of the labor market	19	**30**		26	31	7	16	20	33	16	28
Income as a % of the federal poverty level											
Below 100%	11	**29**	**0.040**	14	36	14	27	8	19	11	31
100%–199%	16	**24**		18	27	19	18	10	33	17	17
200% or higher	73	**22**		67	22	67	15	82	26	71	19
Relationship status											
Married	43	22	0.230	42	26	45	15	42	22	42	19
Cohabiting	21	22		24	22	22	19	15	32	24	15
Formerly married, not cohabiting	4	27		4	29	4	30	3	34	4	17
Never married, not cohabiting	33	25		30	25	29	18	39	28	30	26
Health insurance coverage^d^											
Private	79	**21**	**<0.01**	71	21	84	16	82	25	82	19
Public	13	**28**		16	26	12	25	12	33	12	26
None	8	**33**		12	42	3	38	6	26	7	20
Sexual activity^e^											
Currently sexually active	85	24	0.370	85	26	86	18	83	27	84	21
Not currently sexually active	15	21		15	22	14	15	17	23	16	22
Using provider‐involved contraception^f^											
No	33	**36**	**<0.001**	31	39	28	29	38	41	30	29
Yes	67	**16**		69	18	72	13	62	16	70	16
Receipt contraceptive care in past 12 months^g^											
No	41	**28**	**<0.001**	41	30	33	23	41	35	46	21
Yes	59	**19**		59	22	67	15	59	20	54	19
Receipt of person‐centered contraceptive care^h^											
Less than excellent care	45	**25**	**<0.001**	50	29	40	20	47	26	40	23
Excellent care	55	**15**		50	15	60	11	53	14	60	18

*Note*: Contraceptive users for this analysis include respondents who reported using any method of contraception other than tubal/female sterilization as their only method in the 3 months prior to the survey and who indicated at least one preference for a method of contraception; samples are weighted to reflect women ages 18–44 within each state survey; Race, ethnicity, sexual orientation, income, relationship status, health insurance coverage and provider‐involved contraception use all excluded missing responses.

^a^ Pearson's X^2^ test. Significance is indicated by bolding.

^b^ Includes lesbian, gay, bisexual, pansexual, queer, or other.

^c^ Respondents who were out of work for less than a year or more were considered to be unemployed and those who were retired or a full‐time student or homemaker were considered to be out of the labor market.

^d^ Private insurance includes employer‐based plans and plans purchased on the marketplace or exchange. Public insurance options include Medicaid, Medicare, Tricare, Indian Health Service, and State Family Planning Program. Forty‐one respondents who reported to have “other” insurance were excluded.

^e^ Respondents were considered sexually active if they had indicated having penile‐vaginal sex or sex that could lead to pregnancy in the 3 months prior to the survey.

^f^ Provider‐involved methods include the implant, IUD, tubal ligation, pill, patch, ring, and injection.

^g^ Includes receipt of contraceptive counseling, check‐up, or test related to contraception or a contraceptive method.

^h^ Respondents were considered to have received person‐centered care if they reported having received a contraceptive‐related care visit in the past 12 months and they rated this care as excellent on each of the following four domains: respecting the respondent as a person, letting the respondent say what mattered to them about birth control, taking the respondent's preferences about their birth control seriously, and giving the respondent enough information to make the best decision about their birth control; respondents who had not received contraceptive care in the past 12 months were categorized as having received no care.

Overall, 23% of respondents across states reported preferring to use a method other than their current method, ranging from a low of 17% in Iowa to a high of 26% in New Jersey (Table 1). This preference varied across most sociodemographic characteristics included in this study. About one‐third of respondents across states (30%–36%) who reported being of Black or American Indian/Alaska Native race, identified as Hispanic, were born outside of the United States, self‐identified as not straight, were out of the labor market, were uninsured, or were not using provider‐involved contraceptive methods had an unfulfilled contraceptive preference.

### 3.2 Characteristics associated with unfulfilled contraceptive preferences

In multivariable analysis, after controlling for all sociodemographic characteristics and state context, respondents under 40 on average had probabilities of having an unfulfilled contraceptive preference about 5%–13% higher than those aged 40–44 (CIs 0.4%–20%) (Table 2, Model 1). Non‐cohabiting, formerly married respondents had higher probabilities of unfulfilled contraceptive preferences as compared to their married counterparts (aME = 16.6%, CI 6.0%–27.2%). Respondents who reported using a contraceptive method that did not require a provider's involvement on average had probabilities of unfulfilled contraceptive preferences about 23% higher than those using a method that had some level of provider involvement (CI 18.3%–27.3%).

**TABLE 2:** Adjusted marginal effects and 95% confidence intervals for the relationship between respondent characteristics and having an unfulfilled contraceptive preference among contraceptive users and among contraceptive users who had recently received contraceptive care, pooled across four study states, 2019–2020.

	Model 1, *n* = 4055			Model 2, *n* = 2302		
	Adjusted marginal effects, %	95% CI, %		Adjusted marginal effects, %	95% CI, %	
		LL	UL		LL	UL
State						
New Jersey	Ref			Ref		
Arizona	−2.63	−8.0	2.7	−1.56	−8.1	5.0
Iowa	−5.38	−10.4	−0.4	−2.38	−8.2	3.4
Wisconsin	−3.46	−8.8	1.9	1.66	−5.1	8.4
Age						
18–24	13.13	6.3	20.0	11.64	3.7	19.6
25–29	8.17	2.5	13.8	2.11	−4.5	8.7
30–34	5.42	0.4	10.4	6.05	−0.6	12.7
35–39	6.40	1.5	11.3	3.94	−2.4	10.3
40–44	Ref			Ref		
Race and ethnicity						
White non‐Hispanic	Ref			Ref		
Black non‐Hispanic	7.70	−3.1	18.5	3.79	−9.9	17.4
Multi‐racial or other non‐Hispanic	−4.05	−10.6	2.5	−5.58	−13.0	1.8
Hispanic	3.38	−2.5	9.3	5.06	−2.6	12.7
Nativity						
Born in the United States	Ref			Ref		
Born outside of the United States	5.75	−1.5	13.0	0.58	−8.7	9.9
Sexual orientation						
Straight	Ref			Ref		
Not Straight^a^	5.63	−1.2	12.4	2.76	−5.7	11.2
Educational attainment						
High school graduate, GED, or less	Ref			Ref		
Some college or associate degree	4.23	−2.1	10.5	5.08	−2.0	12.2
College graduate or more	2.09	−4.2	8.4	4.35	−2.8	11.5
Employment^b^						
Employed	Ref			Ref		
Unemployed	4.50	−4.7	13.7	−0.27	−8.7	8.2
Out of the labor market	4.57	−1.2	10.3	9.05	1.6	16.5
Income as a % of the federal poverty level						
Below 100%	2.77	−4.3	9.8	1.12	−7.2	9.4
100%–199%	−2.12	−7.5	3.2	0.34	−6.8	7.5
200% or higher	Ref			Ref		
Relationship status						
Married	Ref			Ref		
Cohabiting	1.69	−3.8	7.2	−1.27	−8.0	5.5
Formerly married, not cohabiting	16.59	6.0	27.2	10.47	−2.4	23.3
Never married, not cohabiting	4.38	−1.0	9.8	−1.84	−7.9	4.2
Health insurance coverage^c^						
Private	Ref			Ref		
Public	4.68	−2.3	11.6	5.83	−3.1	14.8
None	5.21	−2.8	13.2	7.54	−4.0	19.1
Sexual activity^d^						
Currently sexually active	Ref			Ref		
Not currently sexually active	−1.12	−6.5	4.3	−3.96	−10.2	2.3
Using provider‐involved contraception^e^						
No	22.77	18.3	27.3	21.94	12.9	30.9
Yes	Ref			Ref		
Receipt of person‐centered contraceptive care^f^						
Less than excellent care				8.76	3.9	13.6
Excellent care				Ref		

*Note*: Percentages are weighted.

^a^ Includes lesbian, gay, bisexual, pansexual, queer or other.

^b^ Respondents who were out of work for less than a year or more were considered to be unemployed and those who were retired or a full‐time student or homemaker were considered to be out of the labor market.

^c^ Private insurance includes employer‐based plans and plans purchased on the marketplace or exchange. Public insurance options include Medicaid, Medicare, Tricare, Indian Health Service, and State Family Planning Program. Forty‐one respondents who reported to have “other” insurance were excluded.

^d^ Respondents were considered sexually active if they had indicated having penile‐vaginal sex or sex that could lead to pregnancy in the 3 months prior to the survey.

^e^ Provider‐involved methods include the implant, IUD, tubal ligation, pill, patch, ring, and injection.

^f^ Among respondent who had received contraceptive care in the 12 months prior to the survey.

Among respondents who had received contraceptive care in the past 12 months (Table 2, Model 2), the youngest women in the sample (18–24) on average still had probabilities of unfulfilled contraceptive preferences higher than the oldest women in the sample (aME = 11.6%, CI 3.7%–19.6%). Respondents who were out of the labor market had higher probabilities of unfulfilled contraceptive preferences compared to their employed counterparts (aME = 9.1%, CI 1.6%–16.5%). Respondents using contraception that did not require provider involvement and those who did not receive excellent person‐centered contraceptive care had higher probabilities of unfulfilled contraceptive preferences than those who were using provider‐involved methods and who received excellent care (aME = 21.9%, CI 12.9%–30.9% and aME = 8.8%, CI 3.9%–13.6%; respectively).

### 3.3 Method‐specific contraceptive preferences

**FIGURE 1:**
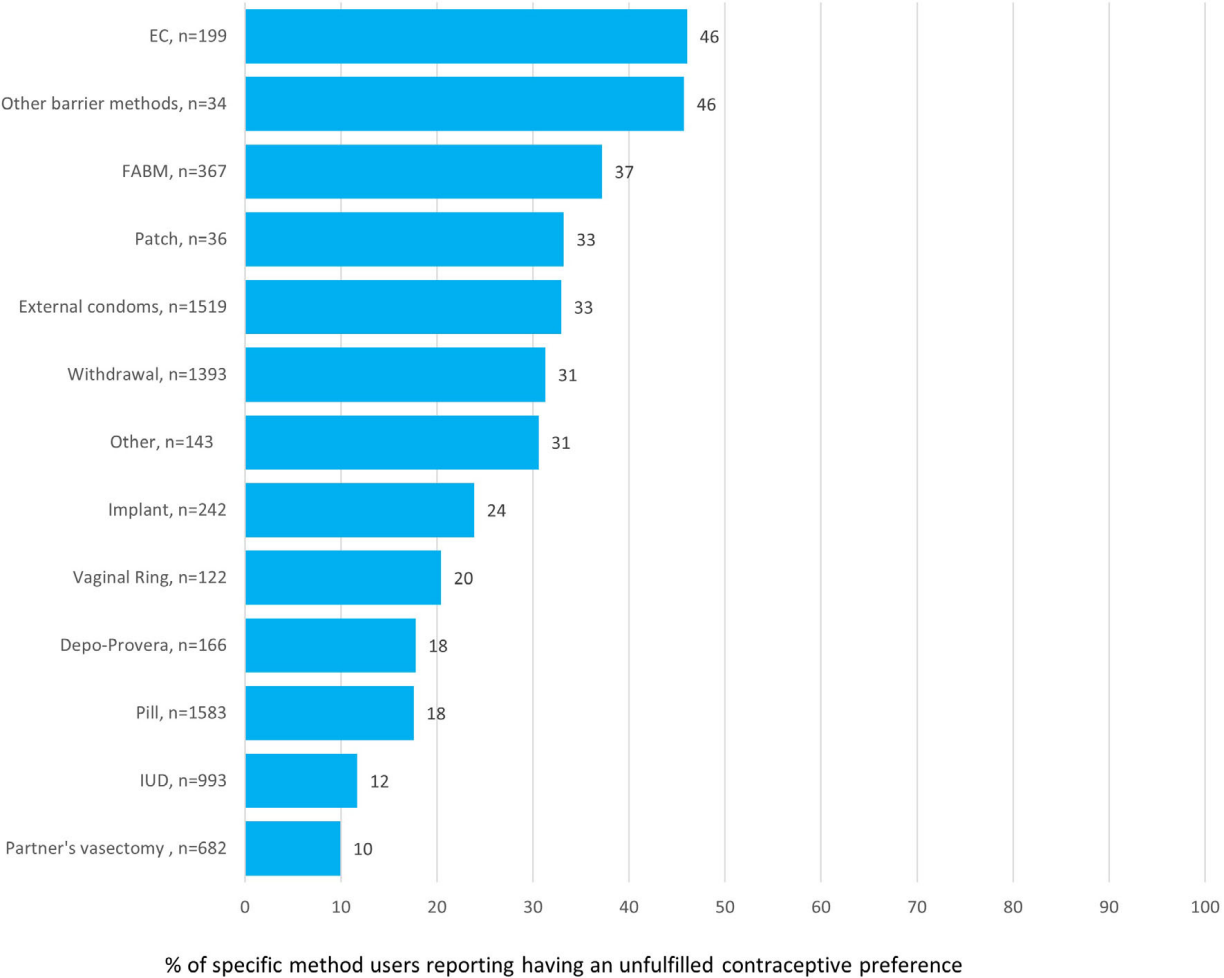
Unfulfilled contraceptive preferences among specific method users.

About one‐third to nearly one‐half of respondents who reported using coital‐specific, barrier, or behavioral methods had unfulfilled contraceptive preferences: 46% each of EC users and other barrier methods users (e.g., diaphragm, internal condom, or sponge), 37% of FABM users, 33% of external condom users, and 31% each of withdrawal and other method users (Figure 1). Eighteen to 33% of respondents using hormonal methods (pills, injections, vaginal rings, patches, and implants) had unfulfilled contraceptive preferences. Respondents using a partner's vasectomy and IUD users, two longer acting or more permanent methods, had the lowest levels of unfulfilled contraceptive preferences (10% and 12%, respectively).

**FIGURE 2:**
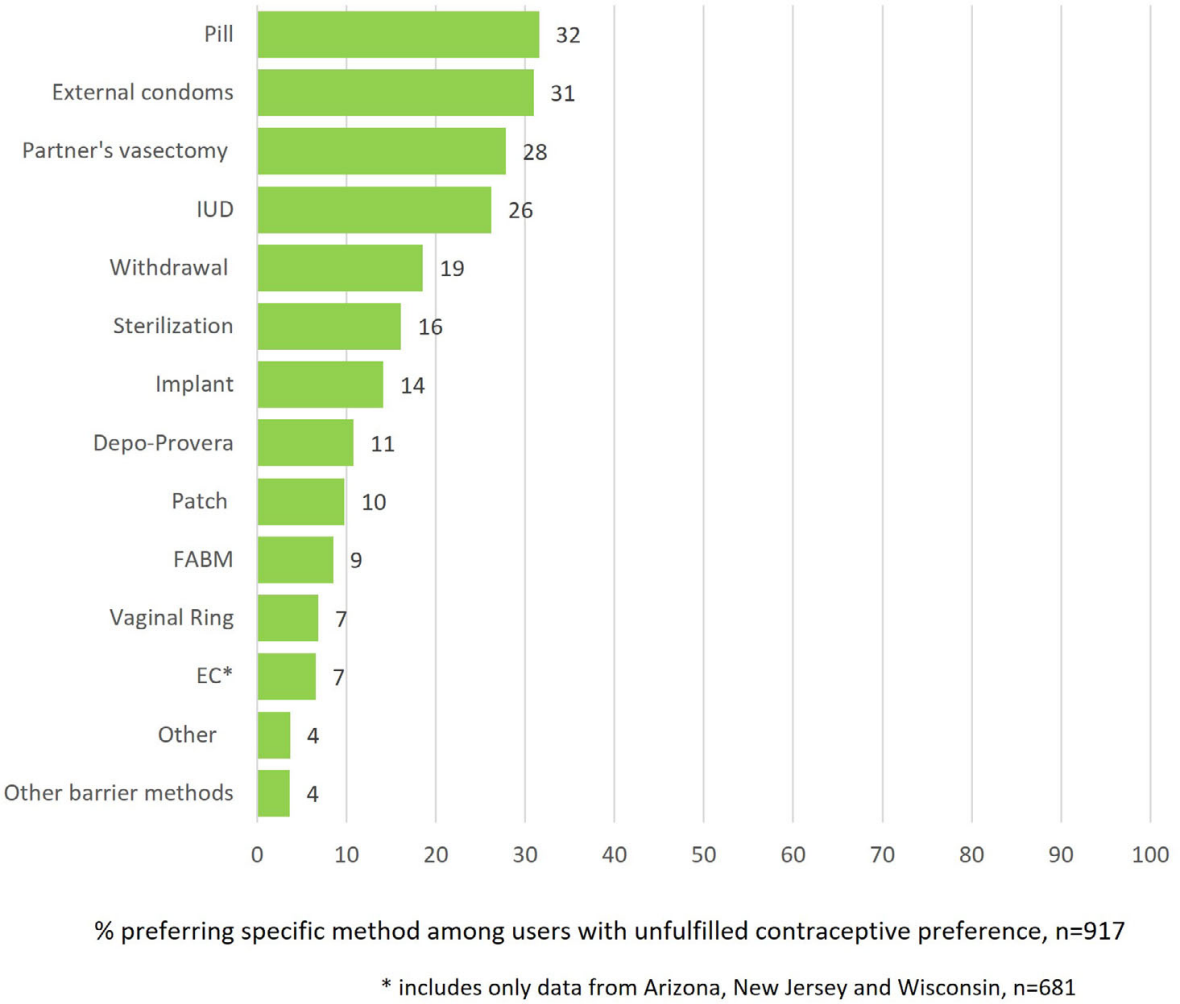
Method‐specific preferences among users with an unfulfilled contraceptive preference.

Among respondents who reported having unfulfilled contraceptive preferences across the four study states (*n* = 917), the most commonly preferred methods were pills (32%), external condoms (31%), partner's vasectomy (28%), and IUDs (26%) (Figure 2). The least commonly preferred methods were other barrier methods (4%), EC (7%), vaginal rings (7%), FABMs (9%), and contraceptive patches (10%).

### 3.4 Reasons for not using a preferred method of contraception

Respondents who had unfulfilled contraceptive preferences cited a variety of reasons for not using their preferred method (*n* = 786), with the most common primary reason falling into either system‐level or interpersonal/individual ones (42% and 41%, respectively) (Table 3).

**TABLE 3:** Reasons for not using preferred method among respondents who had unfulfilled contraceptive preferences.

Reason for not using preferred method of contraception	*N* (Unweighted)	Weighted %
Total	786	100
Systems level reasons	322	42
Cost too high^a^	86	12
Insurance‐related barriers^b^	80	11
Provider‐related^c^	71	9
Access barriers to clinic or method^d^	85	10
Interpersonal/Individual level reasons	347	41
Side effects/health or other concerns^e^	96	12
Infrequent or no penile‐vaginal sex	82	14
Partner/family barriers^f^	116	10
Wants to get pregnant soon or in future	53	5
Other reason^g^	28	3
Not sure	89	14

*Note*: Systems‐level reasons include structural barriers and limitations to obtaining the preferred contraceptive method which includes cost and insurance‐related issues, provider or facility barriers or limitations, and access barriers to method or facility; and interpersonal and individual reasons include individual preferences or concerns or interpersonal barriers.

^a^ Respondents who cite they cannot afford preferred method.

^b^ Respondents who cite not having health insurance, preferred method not covered by current health insurance or cost of insurance copay or deductible is too high.

^c^ Respondents who cite instances when provider advised against method or suggests another method.

^d^ Respondents who cite lack of knowledge on where to get method, difficulty accessing health facility, thier facility does not offer method, fear of lack of confidentiality, or institutional religious barriers.

^e^ Respondents who cite concerns about side effects or health outcomes or contraindications due to health issues. Includes six respondents who reported fear or anxiety around method.

^f^ Respondent's partner or family influences method choice or ability to get preferred method.

^g^ Other reasons include six cases in which respondent's partner has vasectomy or “fixed” but this is not respondent's preferred method; five cases where the respondent cited they did not want to use birth control; 3 cases where the respondent has cited inability to get pregnant. Other reasons also include ambivalence about method, difficulty using method correctly, and recent birth.

About half of the systems‐related reasons behind having unfulfilled contraceptive preferences were due to cost or insurance‐related issues. Provider‐related issues, which include if a provider suggested another method or advised against a preferred method, were cited by 9% of respondents. Access barriers to clinics or methods were cited by 10% of respondents.

About four in 10 respondents reported interpersonal or individual‐related factors as the primary reason for not using their preferred method of contraception. Some respondents reported that they were either infrequently or not having penile‐vaginal sex, while others cited fears of side effects or other health concerns related to their preferred method (14% and 12%, respectively). About 10% reported that their partner or family member influenced their method choice or ability to get their preferred method. Of the remaining respondents citing reasons, 3% had other reasons for not using their preferred method and 14% were not sure why they were not using their preferred method.

## 4 DISCUSSION

Our findings representing the contraceptive preferences of reproductive‐aged women across four states largely support the growing body of evidence in the United States indicating that a sizable minority of contraceptive users are not using their preferred method, and healthcare providers have a role to play in helping people to do so. In addition, we find a persistent association between young age and higher levels of unfulfilled contraceptive preferences, which represents a novel finding that has been documented in only one other larger, population‐based studies examining this outcome.^10^ Our findings on method‐specific preferences among women with unfulfilled preferences highlight the popularity of using a partner's vasectomy, pills, and IUDs as preferred methods. Finally, we highlight how both systems‐level and interpersonal/individual‐level reasons are common factors in whether people are able to realize their contraceptive preferences.

In their recent commentary advocating for contraceptive preference metrics to be more widely adopted as contraceptive indicators, Burke and Potter outline key recommendations for conducting research that incorporates these metrics.^9^ These include asking about method‐specific preferences, delinked from specific conditions, and including a second question asking about reasons for nonuse of preferred method. The contraceptive preference metrics examined in our analysis meet these criteria, supporting the widespread utility of these findings across a variety of state‐based settings. Evidence from our study mirrors that of other population‐based studies at the national and state levels in that approximately 1/4–1/3 of contraceptive users across a variety of state‐ and national‐level studies indicate that they would prefer to use a different method than the one that they are using.^10^, ^11^, ^12^, ^13^ This clear gap between methods used and those preferred to be used highlights that programs and policies designed around simple contraceptive use metrics may be missing a clear opportunity to support greater reproductive autonomy among individuals. Of greater concern is that these programs may be inappropriately prioritizing the recommendation of certain methods over others due to a narrow focus on method effectiveness rather than on the broad range of reasons that individuals consider in their contraceptive decisions.

We find that the four most commonly preferred methods represent a range of types, from hormonal (pills) to coital (condoms) to permanent (vasectomy) to long‐acting yet reversible (IUDs). This diversity of method preferences supports a key tenet of person‐centered care: given the broad range of reasons for which people use contraception^10^ and the diversity of sources people identify for where to obtain it,^22^ no one method fits everyone's needs and individuals should be supported to access their preferred methods. Evidence indicates that even the broad range of currently available methods on the market do not exactly align with features that users identify as ideal,^23^ and our findings document that side effects and/or health‐related factors are a key reason why some people are not using their preferred method. Our findings on preferences around partner vasectomy supplement this evidence and indicate that ongoing contraceptive development to improve current methods as well as expand male contraceptive options may help to address people's contraceptive preferences. Some contraceptive users prefer methods that require less user involvement and contact with healthcare providers, such as IUDs, while others prefer the ongoing maintenance associated with using birth control pills or the nonhormonal and coital‐specific aspect of male condoms.^24^ Finally, that a not insubstantial proportion of respondents indicated that they did not know why they were not using their preferred method, mirroring findings from Ohio,^12^ points to the importance of allowing for ambivalence and fluidity when seeking to understand people's contraceptive desires. This diversity in method preferences supports the importance of regularly incorporating contraceptive preference metrics into the monitoring of family planning and contraception‐focused policies and programming.

Most contraceptive users in this study indicated that they were using their preferred method, and those who had received person‐centered care had higher levels of having fulfilled preferences. Healthcare providers, then, clearly have an important role to play in helping to link patients with their preferred methods, and policies focused on contraceptive care and coverage should be person‐centered. Other research has documented how providers can help mitigate barriers experienced by patients in accessing contraception,^11^, ^25^ while they can also be exacerbators of these barriers.^26^, ^27^, ^28^ For example, given recent evidence indicating that many contraceptive users would prefer to be receiving more information about contraceptive side effects before choosing their method and that health care providers are the preferred source for this information,^10^ contraceptive care providers have a clear mandate to assess patients' contraceptive preferences and to help to fill in this information gap to bring method use and preferences into better alignment. Indeed, a recent study examining experiences of person‐centered contraceptive counseling at the national level highlighted that the lowest‐rated aspect of four contraceptive care domains assessed was related to having their informational needs to make birth control decisions met.^29^ Investing in provider training on patient‐centered best practices for providing contraceptive care is one avenue to pursue to address these gaps.

Beyond providers, access barriers having to do with cost/insurance coverage of contraception are a modifiable lever that could reduce the gap between contraceptive methods used and preferred. Given national evidence that nearly one‐quarter of low‐income contraceptive users would use a different method if cost were not an issue^11^ and the frequency with which both insured and uninsured contraceptive users cite cost as a barrier to using preferred methods in our study and in others,^10^, ^30^, ^31^ enhancing cost coverage via both health insurance and financial subsidies to sexual and reproductive health care delivery remains a vital step for broad contraceptive access. While federal policy via the Affordable Care Act (ACA) intended to address some of these ongoing coverage‐related barriers by mandating no cost sharing for contraceptive methods, more work is needed to close existing loopholes in the ACA that result in out‐of‐pocket costs for contraceptive users; recent guidance released clarifying the birth control benefit under the ACA is a step toward this goal.^32^ In addition, the White House recently announced new actions to strengthen contraception access and affordability for people with private health insurance and to reinforce obligations of insurers—both public and private—to cover affordable contraception,^33^ representing key steps toward closing gaps in contraceptive access.

Young people ages 18–25 use pills and external condoms most commonly among contraceptive methods.^10^ They also report high levels of barriers to accessing contraception^34^, ^35^ and, in our study, even when having recently received contraceptive care, high levels of not using their preferred method. Taken together, this evidence underscores the need for improvements in youth‐friendly contraceptive care, including the importance of confidentiality, having supportive and specialized healthcare providers, and minimizing logistical barriers to accessing contraception.^36^ Young people, in particular, cite side effects as a key reason for not using their preferred method,^10^ and healthcare providers working with youth should adopt person‐centered contraceptive care practices of making sure that contraceptive discussions reflect these patients' unique concerns.

Our study has both strengths and limitations. Data are representative of reproductive‐aged women in Arizona, Iowa, New Jersey, and Wisconsin, and the contraceptive preference metrics examined in our study largely align with recommended best practices for research into people's contraceptive desires.^9^ At the same time, our study does not include data for preferences among nonusers of contraception, leaving much unknown about the extent and types of barriers to contraception that exist for these people and how to meet the contraceptive needs of this population, one that may have increased and different barriers to realizing their preferences related to contraception. Future research should explicitly include nonusers of contraception and ensure that preferences for contraception are elicited broadly and not just within the context of pregnancy prevention reasons, as evidence indicates that people have multiple reasons for wanting to use contraception.^10^ In addition, our study does not include permanent contraceptive users, but other research indicates that this group of users has high levels of fulfilled contraceptive preferences,^13^ potentially indicating that our state‐level metrics of unfulfilled contraceptive preferences may be slightly higher than what would be expected if permanent contraceptive users were included in the sample. Given the explicit naming of “women” in the survey name and “female” in the screener, this study does not necessarily capture contraceptive preferences of nonbinary, trans, and other people who do not identify with this gendered language but who have capacity for pregnancy and contraceptive needs and preferences, thus limiting generalizability of these findings to this full population of interest. Due to the overlap in timing with survey fielding and both the 2019 Title X Final Rule (“domestic gag rule”) and the early months of the COVID‐19 pandemic, contraceptive preferences documented in this study were captured during a time period when multiple, extenuating factors that also impacted contraceptive access were occurring.^37^ Future research should delve into the extent to which these cross‐sectionally documented preferences change over time, and what factors are associated with changes in preferences.

Given the increasing restrictions on access to sexual and reproductive health care in the wake of the 2022 *Dobbs v. Jackson Women's Health* decision, identifying new pathways to ensure access to the full range of contraceptive methods is more critical than ever. Building programs and healthcare delivery around people's preferences for which contraceptive methods to use is an important step toward sexual and reproductive health equity, or ensuring that systems reflect the needs of all people, across the range of age, gender, race, and other intersectional identities, to attain their highest level of sexual and reproductive health.^38^ Our findings highlight that avenues for decreasing the gap between contraceptive methods used and those preferred to be used may lie with healthcare providers and funding streams that support the delivery of contraceptive care.
